# Nanoscale Piezoelectric Properties and Phase Separation in Pure and La-Doped BiFeO_3_ Films Prepared by Sol–Gel Method

**DOI:** 10.3390/ma14071694

**Published:** 2021-03-30

**Authors:** Alina V. Semchenko, Vitaly V. Sidsky, Igor Bdikin, Vladimir E. Gaishun, Svitlana Kopyl, Dmitry L. Kovalenko, Oleg Pakhomov, Sergei A. Khakhomov, Andrei L. Kholkin

**Affiliations:** 1 Faculty of Physics and Information Technology, Francisk Skorina Gomel State University, 246019 Gomel, Belarus; alina@gsu.by (A.V.S.); sidsky@gsu.by (V.V.S.); Vgaishun@gsu.by (V.E.G.); dkov@gsu.by (D.L.K.); khakh@gsu.by (S.A.K.); 2 TEMA, Department of Mechanical Engineering, University of Aveiro, 3810-193 Aveiro, Portugal; bdikin@ua.pt; 3 Department of Physics, CICECO-Aveiro Institute of Materials, University of Aveiro, 3810-193 Aveiro, Portugal; svitlanakopyl@ua.pt; 4 Laboratory for “Electro-and Magnetocaloric Materials and Structures”, ITMO University, 197101 St. Petersburg, Russia; oleg.cryogenics@gmail.com

**Keywords:** bismuth ferrite, La-doping, piezoelectricity, sol–gel

## Abstract

Pure BiFeO_3_ (BFO) and doped Bi_0.9_La_0.1_FeO_3_ (BLFO) thin films were prepared on Pt/TiO_2_/SiO_2_/Si substrates by a modified sol–gel technique using a separate hydrolysis procedure. The effects of final crystallization temperature and La doping on the phase structure, film morphology, and nanoscale piezoelectric properties were investigated. La doping and higher crystallization temperature lead to an increase in the grain size and preferred (102) texture of the films. Simultaneously, a decrease in the average effective piezoelectric coefficient (about 2 times in La-doped films) and an increase in the area of surface non-polar phase (up to 60%) are observed. Phase separation on the films’ surface is attributed to either a second phase or to a non-polar perovskite phase at the surface. As compared with undoped BFO, La-doping leads to an increase in the average grain size and self-polarization that is important for future piezoelectric applications. It is shown that piezoelectric activity is directly related to the films’ microstructructure, thus emphasizing the role of annealing conditions and La-doping that is frequently used to decrease the leakage current in BFO-based materials.

## 1. Introduction

Recently, there has been a rising interest in multiferroic materials, which demonstrate both magnetic and polarization order and resulting coupling between them in a single phase [1,2,3,4,5,6,7]. If an external electric field is applied, multiferroics can change their magnetic moment (converse magnetoelectric effect), and magnetic field can influence dielectric parameters such as electric polarization (direct magnetoelectric effect) or dielectric constant (magneto capacitance). As such, the possibility of manipulating the electric parameters via a magnetic field and may lead to new applications such as magnetic/electric memories, spintronics, magnetocapacitive transducers, magnetic field sensors, etc. Bismuth ferrite, BiFeO_3_ (BFO), is one of the most studied multiferroics having perovskite structure (space group *R3c*) and exceptionally high ferroelectric and magnetic Curie points (830 °C and 402 °C, respectively). The last few years have witnessed a continuing interest in BFO due to the observation of the large conductivity of ferroelectric domain walls and the possibility to manipulate both magnetic and ferroelectric properties by appropriate doping. Weak magnetization of BFO due to antiferromagnetic G-type ordering restricts its application, and there is a chance for magnetization to be increased by doping. One of the biggest problems in BiFeO_3_ films is their large leakage current, which strongly affects both ferroelectric and dielectric properties. One of the disadvantages of BiFeO_3_ films is due to their high conductivity explained by different oxidation states of Fe ions, Fe^3+^ and Fe^2+^, which result in the appearance of oxygen vacancies that are responsible for the hopping conduction [8,9]. Conductivity in BiFeO_3_ films has to be decreased to improve their use in electronic applications. One of the efficient means is to dope them at different sites of the perovskite lattice. Doping of BFO has been extensively studied in the past: there were reports on Tb [10], La [11,12], Ce [13], Eu, Gd, Dy [14] doping for A-site and Ti [8], Cr [15], Zr [16], Mn [17] substitutions at B-site. Using these substitutions it was possible to stabilize the valence of iron with the simultaneous decrease of conductivity. In particular, La-doping was frequently used to create structurally metastable states with both enhanced piezoelectricity and reduced leakage [8,9]. These states, characterized by notable magnetic properties, may occur at the boundaries of nanosized grains and various interfaces in thin films. For example, the bottom electrode’s resistance was found to affect ferroelectric switching kinetics [1]. Magnetic field effect on polarization switching in BFO films was studied in [2]. Magnetic ordering as a function of mechanical strain was found to be important in BiFeO_3_ films [4]. However, no detailed nanoscale measurements of piezoelectric activity in doped BiFeO_3_ films have not been reported yet.

The sol–gel method can be applied to process a variety of materials, including thin films with controlled functional properties [18]. Most of the materials prepared by sol–gel are have improved properties and broadly used in modern technologies [19,20,21]. During the synthesis of multicomponent sol–gel materials, the difficulties associated with different reaction rates for various elements similar to those described in [22,23,24] must be solved. In this work, a modified sol–gel process, in which the initial components are dissolved separately to form homogeneous organic solutions, followed by hydrolysis and polycondensation of the reaction products, leading to the formation of sol, and then to the final colloidal phase was used. The distinctive feature of the technique used in this article is separate hydrolysis procedures for metal (Bi, Fe and La) precursors in order to reach higher chemical homogeneity and to reduce the final crystallization temperature.

## 2. Materials and Methods

BiFeO_3_ (BFO) thin films were synthesized using the standard sol–gel technique. Bi(NO_3_)_3_·5H_2_O) and Fe(NO_3_)_3_·5H_2_O were used as raw reactants with ethylene glycol, dimethyl formamide, and citric were the solvents. All the chemical reagents used were obtained from MilliporeSigma (analytical grade, Munich, Germany). The original feature of the method is the use of separate hydrolysis, that is, each of the metal salts were separately dissolved in the mixture of solvents, kept for 24 h, then the sols were mixed and additionally kept for 24 h. We believe that this additional step allows for more complete hydrolysis and polycondensation reactions and uniform formation of bonds at the molecular level, which will result in the increased homogeneity of the material.

The films were deposited via spin-coating of the final solutions on Pt/TiO_2_/SiO_2_/Si substrates at 2000 rpm (30 s duration). Sol was applied to the substrate using a precision Cee^®^ 200X centrifugal coating device (SPS-Europe B.V., Putten, The Netherlands). Sol (3 mL for 1 step) was poured onto the substrate at the same time. After this, wet layers were dried at 350 °C (4 min), and then thermally processed at 550 °C (5 min in air) by RTA (rapid thermal annealing). This was repeated 3 times to get the required thickness (≈300 nm). Such obtained layers were then annealed at 200 °C for 20 min, 400 °C for 20 min, and crystallized at 550 °C, 600 °C, 700 °C for 20 min, respectively. The average thickness of the coating per application step is 95–100 nm. Bi_0_._9_La_0_._1_FeO_3_ (BLFO) thin films were produced using analogous procedure. Lanthanum nitrate, La(NO_3_)_3_·5H_2_O, was then used for doping. In this case, all metal salts were dissolved separately, including La-nitrate.

X-ray diffraction measurements were done at the University of Aveiro (Aveiro, Portugal) with a Panalytical Empyrean diffractometer (Malvern Panalytic, Almelo, The Netherlands) having Cu Kα1 cathode (λ = 0.15406 nm) and linear PIXEL detector (divergence slit 1/2°). The intensity of the diffractograms was measured by the continuous counting method (step 0.02°, time 200 s) in the 2θ range of 5–90°. Atomic Force Microscopy (AFM) measurements were conducted using a Bruker Multimode instrument (Bruker Nano Surfaces, Santa Barbara) with a Nanoscope (IV) MMAFM-2 unit. The local piezoelectric activity was evaluated using a standard contact mode with Piezoresponse Force Microscopy (PFM) capability. The PFM method is using the inverse piezoelectric effect, which couples electrical and mechanical responses in a sample. The voltage applied to a piezoelectric sample through a conductive tip produces local changes in its dimensions. To detect the polarization distributions the PFM tip is rastered across the film’s surface. Details of the PFM measurement procedure are given in [25].

## 3. Results and Discussion

**Figure 1:**
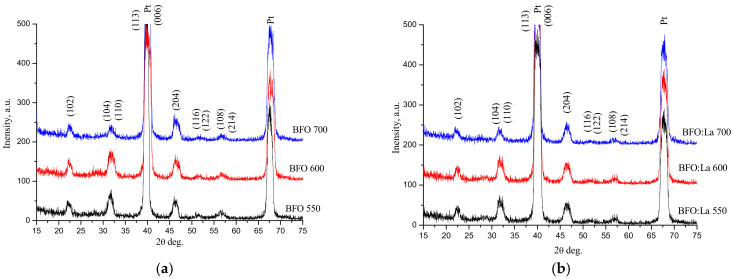
XRD profiles of BiFeO_3_ (**a**) and Bi_0_._9_La_0.1_FeO_3_ (**b**) sol–gel films.

XRD data for pure and doped BiFeO_3_ films (Figure 1 and Table 1) annealed at different temperatures were analyzed. Phase analysis was carried out by comparing the experimental interplane distances d with X-ray patterns of the database of the International Diffraction Data Center ICDD PDF2 [26]. Perovskite phase content was obtained by the calculation of intensity ratios of experimental and analytical peaks using Jana 2006 program [27]. In order to get the best approximation of the intensities of the analytical lines of diffractograms, the grating parameters were refined following the procedure described in [27]. The calculation of crystallite sizes was carried out using the Scherer formula [28]. Rietveld refinement used the experimental XRD profile by accounting for the instrumental contribution with the help of Profex interface (BGMN program) [29]. It was found that BiFeO_3_ films exhibit completely different behaviour as compared to powders [30]. The formation of crystalline structure with high content of a perovskite phase started already at 550 °C. An increase in the synthesis temperature to 600 °C leads to a concurrent increase in the perovskite phase content. Further annealing at 700 °C does not lead to a decrease in the content of the required phase (unlike powders of the same composition) due to possible Bi volatilization [31].

As judged from the XRD measurements for BFO and BLFO thin films (Figure 1, Table 1), the content of the perovskite phase in La-doped films is higher than in pure BiFeO_3_ annealed at the same temperature. This may be explained by the lower degree of volatilization of Bi when it is substituted by La^3+^ [32]. We believe that the addition the lanthanum nitrate leads to a significant decrease in the concentration of intrinsic defects as it was observed in BiFeO_3_ ceramics (see [32] and references therein). Additionally, this results in the formation of perovskite structure at lower temperatures, e.g., 550 °C, for lanthanum-doped BiFeO_3_. The observed increase in the content of the perovskite phase was also observed with increasing annealing temperature from 550 °C to 700 °C for both compounds and La-doped films were found to contain significantly less amount of secondary phases (Table 1). This is an expected result taking into account our previous study of BFO and BLFO phase formation [31] and the fact that La addition should promote better chemical homogeneity while simultaneously resulting in the increase of the crystallite size (Table 1).

In addition, it can be noted that the annealing temperature notably affects the orientation of the grains (i.e., the films’ texture). For the films annealed at 550 °C and 600 °C, the most intensive peak is at 32 degrees, which corresponds to the (110)/(104) planes of the pseudocubic structure. However, for the films crystallized at 700 °C, the most intensive peak becomes (204). The peak intensity ratios of 550 °C and 600 °C annealed films correspond to a random orientation of the grains [33]. Changing these ratios indicates the predominant orientation of the grains with (102) orientation parallel to the surface of the films. The appearance of (012) texture in the films annealed at high temperature (700 °C) is similar to that observed in ref. [33] and can be related to the growth mechanism, rather than to the effect of the substrate.

**Figure 2:**
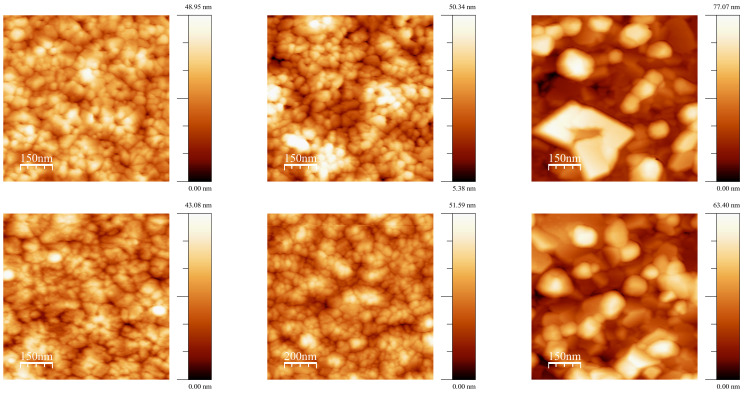
Surface structure of BiFeO_3_ (**top row**) and Bi_0_._9_La_0.1_FeO_3_ (**bottom row**) sol–gel films obtained by AFM at 550 °C (**left column**), 600 °C (**middle column**) and 700 °C (**right column**).

The results of the investigation of the surface morphology of the films after annealing at different temperatures are presented in Figure 2 (AFM images) and summarized in Table 2.

Figure 2 shows the AFM topography images of BFO and BLFO thin films annealed at 550 °C, 600 °C and 700 °C, respectively. The surface morphologies, grain size, geometric shape, homogeneity, and agglomeration of the nanograins can be visualized based on AFM scans. The average grain size was calculated with the Gwyddion program [34]. It can be seen from Figure 2 that, as the annealing temperature increases up to 700 °C, the average grain size increases for both BFO and BLFO thin films. After annealing at 550 °C, the average grain size is about 90 nm, and crystallization at 700 °C results in the average grain size of 100 nm, whereas for some grains it is as large as 300–400 nm (Figure 2). The appearance of relatively large grains for undoped BFO films annealed at 700 °C (Figure 2) is consistent with the observation of increased (102) texture at the same temperatures, so we hypothesize that these grains are (102) oriented. Surface roughness is increased with increasing annealing temperature for both compositions. This is in line with the increased grain size and longer diffusion paths at elevated temperatures. Note that the crystallite size is much less than the grain size and does not depend much on the temperature (except for BFO films annealed at 550 °C with a large amount of secondary phase). It means that the chemical homogeneity within the grains is more or less the same at different annealing temperatures, suggesting that the final crystallization anneal can be done at low temperatures (600 °C).

**Figure 3:**
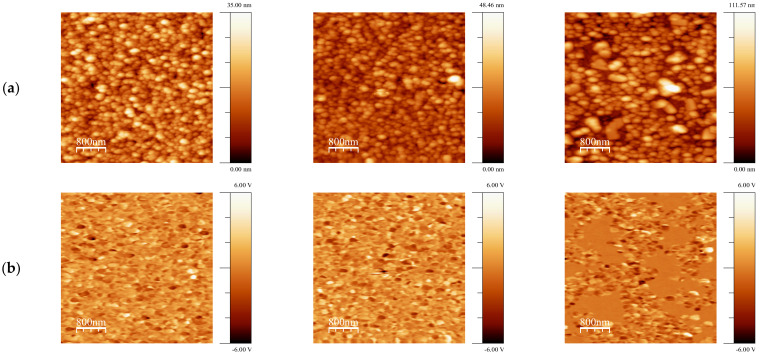
(**a**) Topography, (**b**) out-of-plane (OPP) Piezoresponse Force Microscopy of BiFeO_3_ sol–gel films annealed at at 550 °C (**left column**), 600 °C (**middle column**) and 700 °C (**right column**).

**Figure 4:**
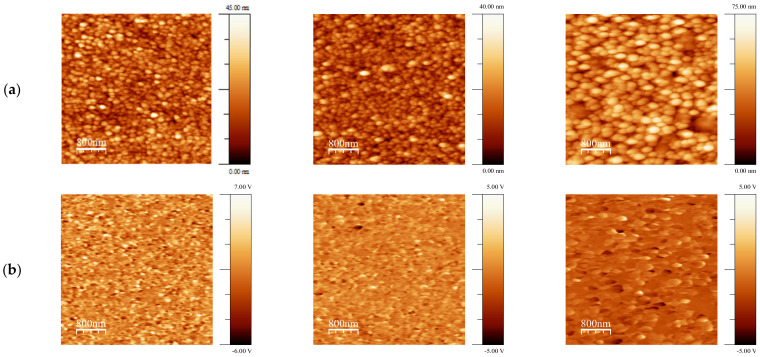
(**a**) Topography, (**b**) OPP PFM of Bi_0_._9_La_0.1_FeO_3_ sol–gel films annealed at 550 °C (**left column**), 600 °C (**middle column**) and 700 °C (**right column**).

**Figure 5:**
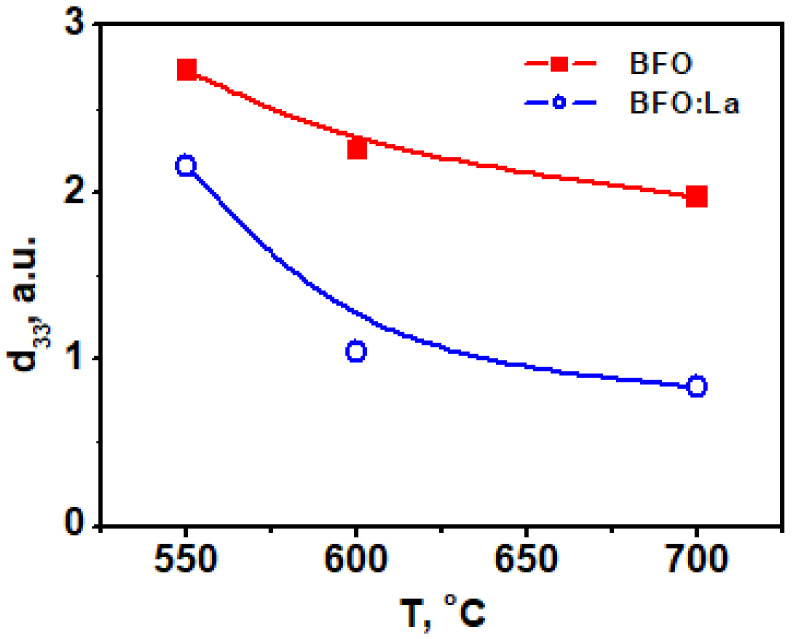
Evolution of the effective piezoresponse of BFO and BFO:La films annealed at different temperatures.

**Figure 6:**
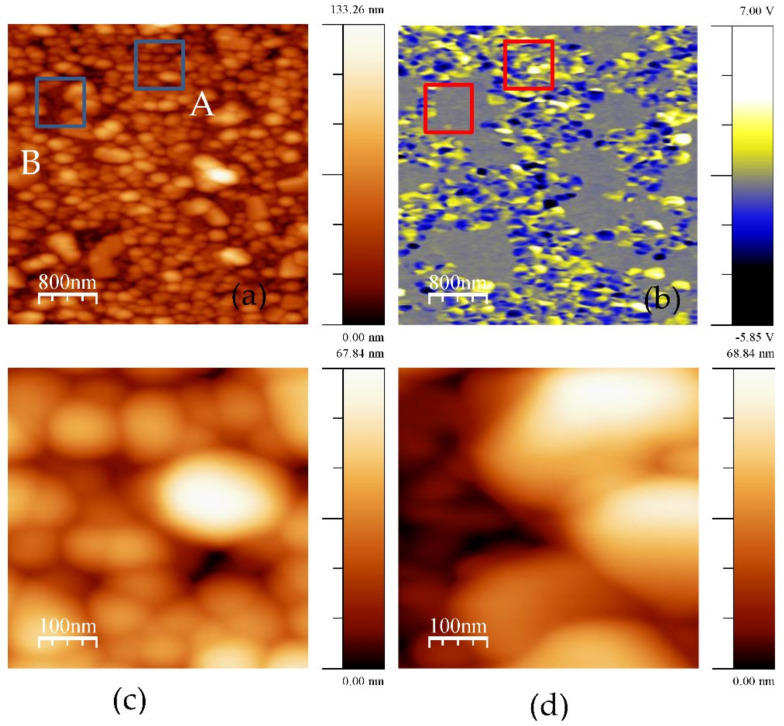
BFO film annealed at 700 °C: (**a**) Topography, (**b**) piezoresponse; and zooms of areas A (**c**,**d**) shown in Figure 7a. Estimated average grain size of A is 100 nm and B—300 nm.

In order to understand the suitability of deposited BFO-based films for piezoelectric applications PFM method was applied to visualize not only the grain morphology but also their local piezoelectric activity. For grains, in which the polarization is normal to the film’s plane, the amplitude of piezoelectric deformation ε measured by PFM is proportional to the longitudinal (or vertical) effective d_33_ coefficient and its phase (close to 0 or to 180°) is a measure of the polarization direction: ε_3_ = d_33_ × E_3_ in (*hkl*) grain and −ε_3_ = d_33_ × (−E_3_) in (-*h-k-l*) grain. The 0-degree phase corresponds to the “positive” polar axis and “positive” piezoresponse. If the phase shift is 180°, it indicates the “negative” orientation of the grains and the “negative” piezoresponse. In Figure 3 and Figure 4, the so-called mixed PFM signal is used, in which the phase is reflected in the signal sign. Vertical or out-of-plane (OPP) was measured, which is proportional to the effective piezocoefficient d_33_ [35]. These images are shown for BFO and BLFO thin films annealed at different temperatures. A convenient way to describe the piezosignal distribution is to plot it in the form of histograms showing the strength of the signal as a function of the number of pixels in the entire image [36]. In this way, a complete polarization distribution in as-grown (i.e., not poled by the external electric field) films can be obtained. We note that, due to the small grain size, the contrast is approximately constant within the grain, and no ferroelectric domains are observed. The histograms of the OOP signals taken from the images in Figure 3 and Figure 4 are shown in Figure 5. Comparing the widths of these distributions, one can qualitatively estimate the evolution of piezoelectric activity in the films as a function of annealing temperature (Figure 5 and Figure 6).

Figure 5 features a clear decrease in the piezoelectric signal with annealing temperature for both doped and undoped BFO films. In general, doped films are less piezoelectric (Figure 5) because the end member of the solid solution LaFeO_3_ is centrosymmetric [37].

An interesting observation is that some parts of the films show a zero piezoresponse, i.e., they are not piezoelectric at all. These non-polar or antipolar phases (especially *Pnma* phase) have zero polarization and thus give a zero piezoresponse [38]. Another explanation is the appearance of second phases, for example, Bi_2_Fe_4_O_9_, in which polarization should be zero as well [39]. The location of the second phases is most likely on the surface of the films (see [40] and references therein). That is why the relative area of non-piezoelectric inclusions might be higher than the volume concentration of the second phases estimated by XRD. It should be noted that in these areas (where piezoelectric signal is close to zero), the morphology of the grains is different, implying a different growth mechanism. Non-piezoelectric grains are notably larger and the effect is somewhat similar to La addition (Figure 6).

**Figure 7:**
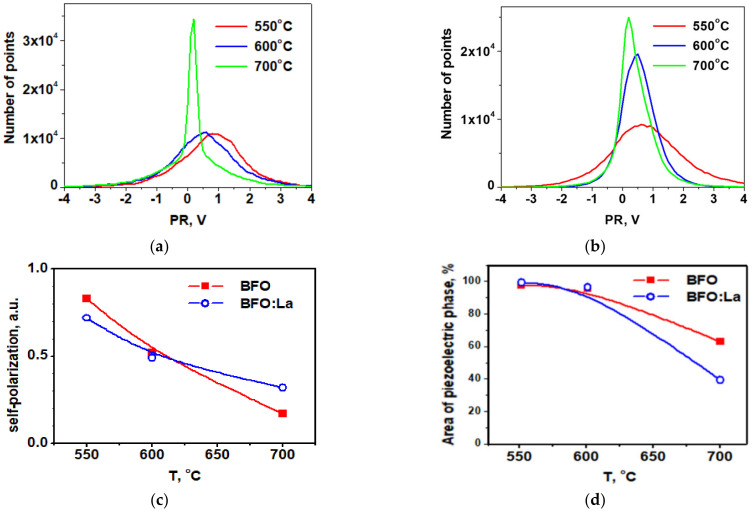
Histograms of the piezoresponse images of BFO (**a**) and BFO:La (**b**) thin films annealed at different temperatures; (**c**) self-polarization in BFO and BFO:La films vs. temperature; (**d**) evolution of areas with piezoresponse after different annealing temperatures.

An important property of ferroelectric films is self-polarization, i.e., the ability of the films to exhibit piezoelectric response without any poling [41,42]. In the PFM technique, it is calculated as the relative difference between the “positively” and “negatively” oriented grains/domains on the surface of the films [43]. If the film is strongly self-polarized it can be used as a sensor or actuator without any dubious poling process, which is very difficult to do in the case of microelectromechanical systems (MEMS) [44]. Figure 7a–c shows the evolution of self-polarization with crystallization temperature and La doping. While it decreases with annealing temperature it never goes to zero because T_c_ in BiFeO_3_ is higher than the crystallization temperature used in our experiments. It means that the model of polarization by the surface barrier (reported in ref. [40]) can be valid. The effect of La signifies that the Schottky barrier height effectively increases with La doping for the films annealed at higher temperatures. For lower temperatures, this effect is much weaker and might be related to a weaker effect of oxygen vacancies generated by La ([38,45]).

Figure 7d demonstrates that the phase separation is apparently facilitated by La addition. If La concentration in non-piezoelectric grains is greater than 15%, the absence of polar properties can be explained by the appearance of *Pnma* non-polar phase [46]. It can be assumed that during annealing, partial phase separation occurs and the sample is actually a composite comprising polar and non-polar grains similar to that observed in rare-earth-doped BFO ceramics [38]. Figure 7d shows that the large parts of the films do not have a piezoelectric signal after annealing at 700 °C, so 60% of the film surface is not piezoelectric. However, X-ray diffraction measurements based on the comparison of the intensity of the major peaks do not show significant changes (Table 1). Thus PFM is proved to be a very sensitive tool for the monitoring of non-piezoelectric phases on the surface.

## 4. Conclusions

Pure BiFeO_3_ and Bi_0.9_La_0.1_FeO_3_ thin films were prepared on Pt/TiO_2_/SiO_2_/Si substrates by a modified sol–gel technique using a separate hydrolysis procedure. The effects of final crystallization temperature and La doping on phase structure, film surface quality, and nanoscale piezoelectric properties were investigated. Major conclusions of the work can be formulated as follows: (i) La doping and higher crystallization temperature lead to the increase of the grain size and apparent (102) texture of the films. (ii) Simultaneously, a decrease in the average effective piezoelectric coefficient (about 2 times in La-doped films) and an increase in the concentration of surface non-polar phase (up to 60%) are observed. (iii) Phase separation on the surface is related to either the appearance of second phases due to Bi loss at the surface or to the formation of the pseudocubic perovskite phase. (iv) As compared with the undoped BiFeO_3_, La-doping also increases the average grain size and self-polarization that is important for future piezoelectric applications. Piezoelectric property is shown to be directly connected to the films’ growth conditions and doping, emphasizing that both should be thoroughly controlled in order to use BFO-based films in micromechanical applications.

## Figures and Tables

**Table 1:** Phase purity and crystallite sizes of BiFeO_3_ and Bi_0.9_La_0.1_FeO_3_ thin films annealed under different conditions.

Sample	Crystallization Temperature, °C	Perovskite Phase Content, % (±2%)	Crystallite Size, nm (±0.5 nm)
BFO	550	70	9
	600	94	5
	700	94	5
BLFO	550	92	5
	600	95	6
	700	97	6

**Table 2:** Surface parameters of BiFeO_3_ and Bi_0_._9_La_0.1_FeO_3_ sol–gel films annealed at different temperatures.

Sample	Ra, nm	Average Grain Size, nm	Sample	Ra, nm	Average Grain Size, nm
BFO 550 °C	8	90	BLFO 550 °C	4	85
BFO 600 °C	5	80	BLFO 600 °C	4	85
BFO 700 °C	10	100	BLFO 700 °C	9	135

## Data Availability

Not applicable.
